# The Impact of Digital Coaching Intervention for Improving Healthy Ageing Dimensions among Older Adults during Their Transition from Work to Retirement

**DOI:** 10.3390/ijerph20054034

**Published:** 2023-02-24

**Authors:** Sara Santini, Paolo Fabbietti, Flavia Galassi, Alessandra Merizzi, Johannes Kropf, Niklas Hungerländer, Vera Stara

**Affiliations:** 1 Centre for Socio-Economic Research on Aging, IRCCS INRCA-National Institute of Health and Science on Aging, 60124 Ancona, Italy; 2 Unit of Geriatric Pharmacoepidemiology and Biostatistics, IRCCS INRCA-National Institute of Health and Science on Aging, 60124 Ancona, Italy; 3 Salumentis OG, 1130 Wien, Austria; 4 AIT, 1130 Wien, Austria; 5 Model of Care and New Technologies, IRCCS INRCA-National Institute of Health and Science on Aging, 60124 Ancona, Italy

**Keywords:** digital coaching intervention, healthy ageing, older adults, retirement, transition to retirement

## Abstract

Retirement is a critical step in older adults’ lives, so it is important to motivate them to stay physically active, mentally healthy, and socially connected in the transition from work to retirement, including through digital health coaching programs. This study aims to: evaluate the impact of a digital coaching intervention to enhance three healthy ageing dimensions, i.e., physical activity, mental well-being, and socialization of a group of adults near retirement; understand the users’ experience; and identify the system strengths and weaknesses. This longitudinal mixed-methods study, carried out in 2021 in Italy and the Netherlands, enrolled 62 individuals. In the first 5 weeks of the trial, participants used a digital coach with the support of human coaches, and then they continued autonomously for another 5 weeks. The use of the digital coach improved the participants’ physical activity, mental well-being and self-efficacy during the first period and only the physical activity in the second. An effective coaching system should be flexible and attractive. High levels of personalization remain the golden key to aligning the health program to the physical, cognitive and social status of the intended target, thus increasing the user-system interaction, usability, and acceptability, as well as enhancing adherence to the intervention.

## 1. Introduction

We are witnessing the rapid ageing of the world population. The extension of life expectancy often means a prolonged disability condition in later life that is placing questions on long-term care systems across the world. The ageing of the population also entails the transition of many older adults from the status of workers to that of retirees. The literature now agrees that retirement is a critical step in people’s lives, full of opportunities and, at the same time, fraught with dangers [1,2]. In fact, alongside the greater availability of free time and the opportunity to use it in many activities, e.g., cultural and physical, the loss of meaningful relationships and interests can creep in, forcing older individuals to re-organize their daily schedule, re-negotiate their roles in the family and in society, and re-design, at least partly, their own identity [3]. 

Thus, the transition to retirement can represent the tipping point for healthy ageing or its opposite, based on the individuals’ response to this existential change. Healthy ageing is defined as “the process of developing and maintaining the functional ability (i.e., people’s capabilities of being and doing what they have reason to value) that enables well-being in older age” [4], whose main predictors are physical activity, mental well-being, and social participation [5,6]. 

There is a lack of consensus on how the retirement transition can affect older adults’ physical activity [7,8,9,10]. On the one hand, according to the activity theory, retirement may be an opportunity to increase physical activity. On the other hand, the literature underlines that during the retirement transition [11], there could be a fluctuation in the levels of intensity of, and in the motivation to do, physical activity, and a change in their types, which may be affected by gender as well. For example, according to a recent qualitative longitudinal study, the main driver of physical activity seems to be body shaping among men and socialization [12] among women. 

Retirement can also influence individuals’ mental well-being and socialization. In fact, weaker and rarer meaningful social relationships, or a change in self-image, self-esteem, and self-efficacy [3] due to the end of the working life, can lead to social isolation, emotional discomfort, and depression [13].

Considering the above, it is very important to motivate older adults in the transition to retirement to stay physically active, mentally healthy, and socially connected so that they can age healthily and re-shape their own role in society without negative repercussions on their physical and mental health.

Health coaching interventions led by human coaches can motivate older adults to have healthier lifestyles in later life, for example, by eating more vegetables, practicing physical activity, and reducing cigarette smoking. Such programs often target individuals with chronic diseases and are aimed at helping patients determine and work toward their goals so that they become more compliant with the health program [14,15,16,17,18]. Health coaching programs, regardless of whether they are led by humans and/or by digital systems, mainly address older adults with chronic diseases, and only a few of them are conceived from a preventive perspective, targeting older adults in good health conditions and/or in transition to retirement [19]. On the contrary, healthy ageing strategies, including, for example, self-management capabilities, goal setting, personalised feedback and health literacy, as well as emotional support, reminders and alerts, could be highly effective for such a group of older adults, especially if in transition to retirement. Nevertheless, such strategies cannot often be managed by healthcare professionals because the available workforce cannot be devoted to education and prevention, being on the frontline of assistance to older patients with acute and chronic care needs. Thus, digital coaching technologies can offer an innovative way to overcome this lack of assistance and care as dialogue systems that sense relevant context, determine users’ intent, and provide feedback to improve users’ overall quality of life [20]. Therefore, digital coaching systems could be any computer program that supports spoken, text-based or multimodal conversational interactions with humans, such as personal digital assistants, virtual personal assistants, conversational agents or chats [21], reinforces and sustains healthy behavioural habits, and continuously monitors daily activities. There is still scant literature on the experience of older adults with digital coaching systems, but recent research showed that relatively young older adults may perceive digital coaching as a potential to motivate them towards physical activity by providing instructive information and motivational feedback [22]. Unfortunately, the efficacy of personal health coaching systems for adults over 55 remains unclear, and few examples are mapped in the field for older adults and even less for older people in transition from work to retirement [19,20]. This study is part of the AgeWell project aimed at co-designing and developing a digital coaching system (hereafter also referred to as Digital Coach-DC) targeting older adults aged 55 and over in the transition from work to retirement. The study aims to evaluate the impact of a digital coaching intervention to enhance physical activity, mental well-being, and socialization as three healthy ageing dimensions and help a meaningful retirement process as a factor influencing healthy ageing. It represents a novel contribution to assessing the value of digital coaching systems in a target population often overlooked by research on the development of this kind of technical solution. The purpose of the study was to answer three research questions: (1) To what extent can the AgeWell digital coach improve physical activity, mental well-being, social networking and the retirement process of older adults switching from work to retirement? (2) What was the experience of users with the digital coach? (3) What are the strengths and weaknesses of the system?

## 2. Materials and Methods

### 2.1. The System

**Figure 1:**
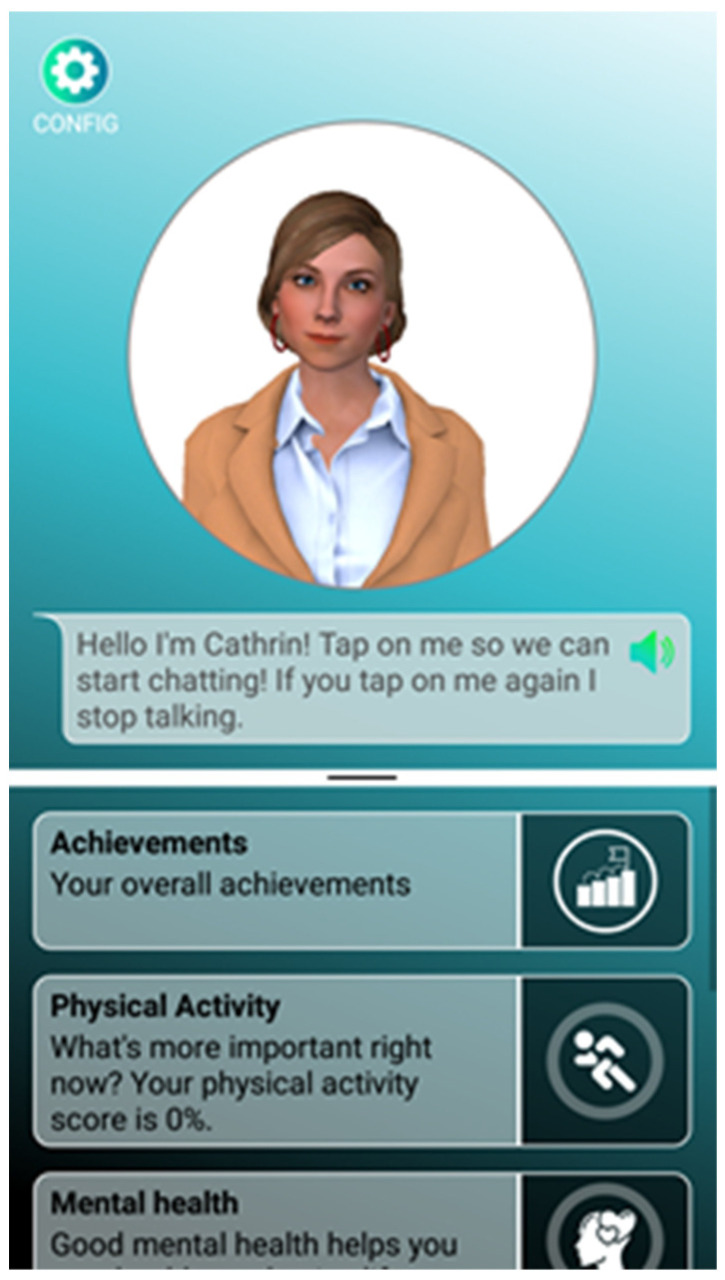
Home of the avatar-based app.

The main aim of the system was to improve users’ healthy ageing attitudes and behaviours by providing them with a series of information and activities grouped into four areas: 1. physical activity; 2. social life; 3. emotional well-being; 4. good retirement. The system was tested as a mobile application (Android App) on a smartphone. For the study, participants were equipped with appropriate devices (Android Smartphone with Android version 8 and upwards), or they used their own devices when preferred and when the user’s private smartphone fulfilled the system’s technical requirements. The software used for the trial consisted of an avatar-based frontend (see Figure 1 below) running on a smartphone and a server component providing the content and collecting the data. Communication between the frontend and the backend was established over the Internet, using secure communication channels with an event-based technology (MQTT—Mosquitto MQTT Server v1.6.8 (Eclipse Foundation, Inc, Ottawa, ON, Canada) following MQTT protocol v3.1.1). The backend server ran within the facilities of one of the project consortium partners, to ensure privacy and security. No personal data were stored in the cloud or with third parties.

**Figure 2:**
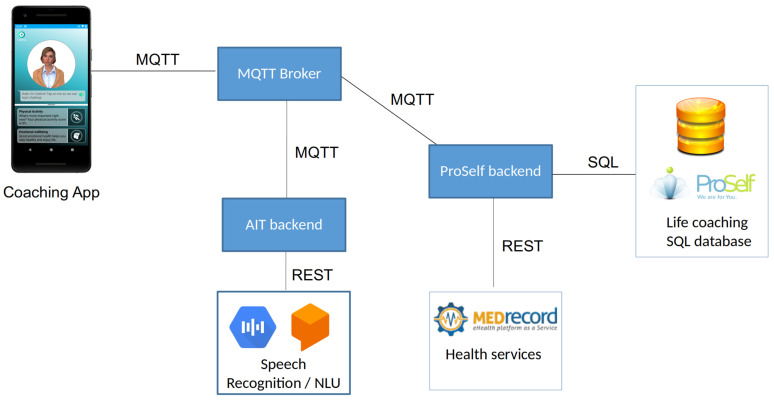
Overall components of the system.

**Figure 3:**
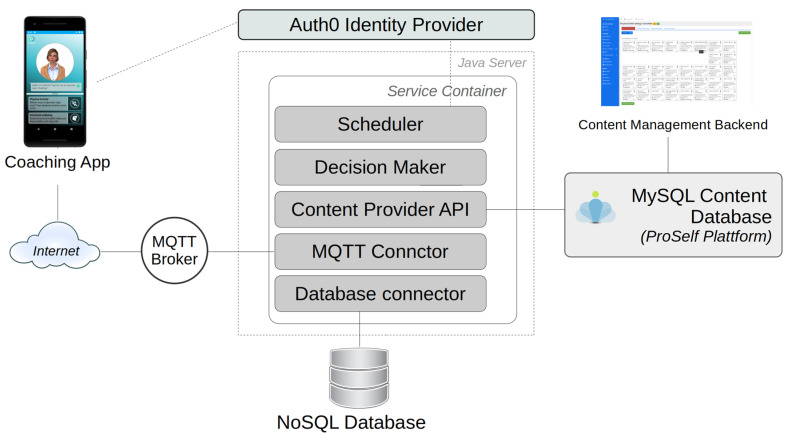
Components of the AgeWell server.

Authentication was implemented using an OAuth2 broker service (www.auth0.com, accessed on 15 March 2020). The server components (see Figure 2 below) were implemented using the Karaf Java OSGi Framework and two databases: a NoSQL database (MongoDB, v3.6.8, MongoDB Inc., New York City, NY, USA) was used to store the user data gathered during App usage and to maintain the user preferences. In contrast, a SQL database (MariaDB, v10.1.29, MariaDB Corp., Espoo, Finland) (Eclipse Foundation, Inc, Ottawa, ON, Canada) was used for static content, e.g., the activities provided via the app. The server components ran on a Linux Virtual Machine (Ubuntu 18.04) (see Figure 3 below). 

The digital coach was co-designed with users through a user-centred design methodology that shaped the realization of four main functional areas stimulating and motivating older adults nearing retirement to improve their physical activity, mental well-being, socialization, and retirement process, as extensively reported in the Supplementary Materials (Table S1).

Among the physical activities to choose from, the app included: walking, tennis, swimming, dancing, and many others. The avatar asked users to choose the preferred activity to be inserted in a weekly plan and to decide on a personal goal. Thereafter, it sent motivational messages to help users reach such a goal, e.g., “Why stop now? You have already achieved so much” or “Once you really get into an exercise schedule, it just gets easier!”, and “Congratulations on making the commitment to exercise! Your body will thank you for it!”

The mental well-being function included three sub-functions: “Mind and body”; “Thought and action”; “Emotions and memories”. In the realm of “Mind and body”, the avatar proposed activities that promoted psycho-bodily well-being and were accompanied by links and/or video tutorials that facilitated their execution. They dealt with the ability to relax, calm down or even find dormant energies through meditation exercises or even more creative or expressive activities such as dancing and singing.

In the sub-realms of “Thought and action”, there were suggestions for activities that stimulated the subject to make plans and stick to them, to put themselves at the centre of their lives, or to remain flexible and see things from different points of view, e.g., the activity named “Bite your tongue!”, which invited users not to complain and pay attention to the positive events of their life.

Finally, in the sub-realm called “Emotions and memories”, there were some suggestions for activities that connect the person to his or her memories and ultimately give a sense of meaning and significance to one’s life, e.g., “Spend some time thinking about your success!” or “Strengthen the relationship with yourself!”.

The system also included activities for stimulating users’ new social contacts and improving the quality of their relationships, e.g., “Making new contacts!” and “Increase the quality of your friendships!”

Finally, the “Good retirement” function included three sub-functions: “Activities for retirement”; “Suggestions” on how to make retirement more meaningful; and “Information on retirement”. In this case, the avatar suggested specific activities that could help people leave the workplace in a healthy way, such as having a retirement party with colleagues or passing on to other younger colleagues their experience gained from years of work, as well as advice on how to garner information about the rights of retirees or even about the activities and facilities offered by one’s municipality of residence.

### 2.2. Study Design

In order to answer the three research questions, the study followed a mixed-methods design in which both qualitative (QUANT) and quantitative (QUAL) data were collected at three measurement times during the 10-week field trial. A concurrent equal status interactive mixed-methods research was adopted, where the QUANT and QUAL data components collection and analyses were executed simultaneously, having, therefore, the same value and being in constant interaction [23,24,25,26,27]. Two parallel QUANT and QUAL strands were integrated into meta-inferences after separate analyses were carried out. Thereafter, QUANT and QUAL results were brought together in the overall interpretation. The QUANT analysis was aimed at highlighting the effects of the digital coach on the physical health, mental well-being, socialization and retirement transition of participants (research questions 1 and 2). The QUAL analysis aimed at shedding light on the strengths and weaknesses of both the digital coach contents and technical aspects (research question 3). The point of integration of the two analyses was reached when the QUAL findings were added and integrated into the QUANT in order to explain to what extent the digital coach influenced users’ lifestyles and how much such an influence can be attributed to the system’s weaknesses and strengths.

**Figure 4:**
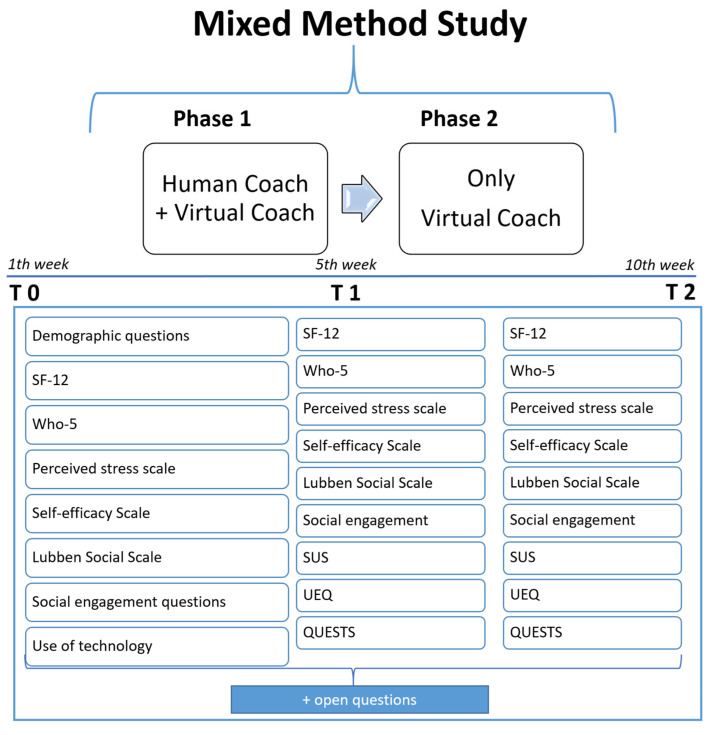
Study design.

As shown in Figure 4, in the first 5 weeks (Phase 1), participants were trained to use the digital coach by two human coaches, i.e., facilitators who constantly offered motivation, supported users’ confidence with the technology, provided technical skills and knowledge, and were responsible for the engagement of users. After the first 5 weeks (Phase 2), study participants continued to use the system without any help from the human coach. They could ask content-related questions and point out technical issues to their coaches via WhatsApp in Italy and via email in the Netherlands. 

During the 5th and 10th weeks of usage, the human coaches administered two online group sessions for gathering users’ impressions about their experience with the system and three online questionnaires on system usability. 

Since the COVID-19 pandemic was at the end of its second wave when the study started (May 2021), the study protocol was adapted in accordance with the global health crisis and the social restrictions for limiting the spread of the virus in the countries where the study was conducted. Thus, online questionnaires were adopted, and the link to them was sent to respondents by email instead of face-to-face administration. Moreover, several questions were added, e.g., on being infected with the virus and self-perception of the impact of the outbreak on individuals’ physical and mental health, in order to bring such variables under control as potential confounding factors. 

The study started with the selection and enrolment of participants in every pilot site (Section 2.2.1). Then, the enrolled individuals were asked to answer an online questionnaire at baseline, after 5 weeks, and after 10 weeks of use of the technology. Along with the pilot, participants were also asked to attend three online group sessions led by a human coach, during which they were asked to answer some open-ended questions to describe their experience with the technology (Section 2.2.2). Then, the data collected were analysed, with QUANT data being analysed statistically and QUAL data being analysed thematically (Section 2.2.3). 

#### 2.2.1. Inclusion Criteria and Recruitment Procedure

**Figure 5:**
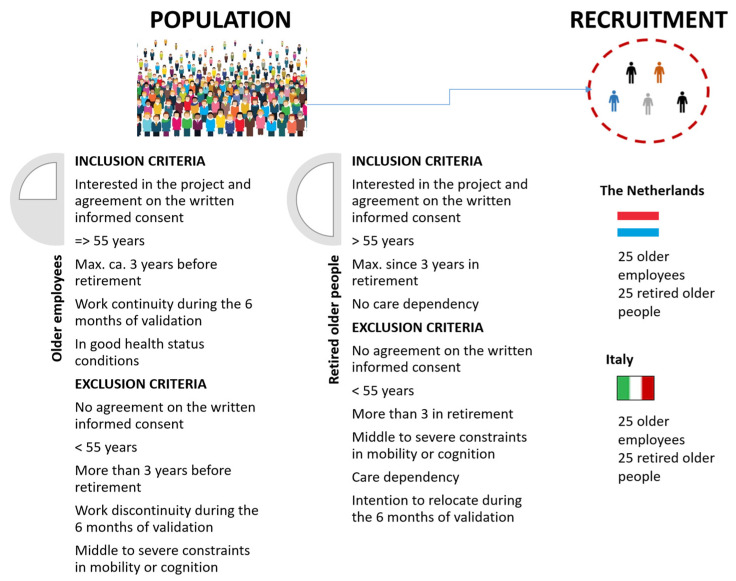
Recruitment Procedure.

According to the AgeWell project activities, a sample of 100 participants was planned to be enrolled (50 in Italy and 50 in the Netherlands). Participants were contacted by phone in Italy and by email in the Netherlands, and they were screened through a battery of questions to check whether they met the inclusion criteria, i.e., being 55 or older, being three years before or three years after retirement, and feeling physically and cognitively healthy (see Figure 5 below). Although the statutory pension age is very similar in the two countries, i.e., 67 years in Italy and 66 years and four months in the Netherlands, without gender differences [28], the participants’ inclusion criteria were quite wide to also include all individuals who were exceptions to the general rule for different reasons, such as because they did demanding jobs such as those in the health sector or because the governments put in place extraordinary measures to facilitate the early exit from the labour market of certain workers’ categories.

In Italy, users were recruited through the research organization’s Human Resources office, voluntary associations and NGOs, private companies, and word of mouth. In the Netherlands, users were contacted by means of the NGO members carrying out the trial and other partner associations from the voluntary sector.

The individuals who agreed to take part in the study were provided with the informed consent form sent via email. Participants were asked to carefully read it, sign it for acceptance, and return it to the researchers by email. The two organizations involved in the trial evaluations applied for ethical approval by the respective competent ethics committees.

#### 2.2.2. QUANT and QUAL Data Collection Tools

In line with the study design, across the trial, life data were collected three times by questionnaires and twice by focus groups, whose tools are described in the following sub-paragraphs together with the analysis methods adopted. Thus, three questionnaires were drafted, one for every evaluation, i.e., before (T0, the baseline), at mid-term (T1, the 5th week of usage), and after the conclusion of the program (T2, the 10th week of usage). The questionnaires, besides demographic questions, consisted of three sections. The first section of the questionnaire focused on physical activity and well-being; the second focused on social life; and the third focused on participants’ experience with the technology. The first two sections of the second and third wave questionnaires (T1 and T2) were the same as the first wave (T0), except for the section on the technology that did not explore the experience with the ICT as at the baseline but instead delved deeper into users’ experience with the digital coach by means of two tools described in the paragraph headed “Quantitative outcome variables and data analysis”. 

The structure of the group sessions with elders in both the study countries foresaw three meetings across the 10-week trial: before the trial, at the middle point, and one week after the end of the experimentation. The first meeting was aimed at training participants in the use of the system, and the other two were aimed at gathering their feedback on their experience with it in daily life as well as their reactions to its functions, usefulness and usability. Due to the pandemic, the meetings with the human coaches took place online. 

In the first meeting, the human coaches showed the system functionality by sharing PowerPoint documents, videos, and smartphones running the system. Participants were also invited to try an activity together, e.g., a short demonstration of a mindfulness and body connection exercise as suggested by the system. In the second meeting, the human coaches asked participants how they were getting on with using the app and whether they had any technical problems, and they made room for further explanations to optimize the use of the system. At the third meeting, the human coaches led a focus group aimed at collecting users’ opinions on the system contents, technical aspects, impact on general health and well-being and suggestions for improvement.

The outcome variables answering the first research question are the SF-12v2 Health Survey [29] to measure the respondents’ physical health; the International Physical Activity Questionnaire (IPAQ) [30] to register the level of physical activity across the digital coach usage; the WHO-5 scale [31,32] and the General Self-Efficacy Scale (GSE-6) [33] to assess mental well-being; and the Lubben 6-item Social Network Scale (LSNS-6) [34,35] to measure the level of socialization.

The SF-12v2 Health Survey [29] is a 12-item subset of the SF-36v2. It is a brief, reliable measure of overall health status through two sub-scales of physical health (PCS) and mental health (MCS). Scores range from 0 to 100, with higher scores indicating better physical and mental health functioning. A score of 50 or less on the PCS-12 is recommended as a cut-off to determine a physical condition, and a score of 42 or less on the MCS-12 may indicate ‘clinical depression’ [36].

The IPAQ [30] identifies three levels of physical activity based on frequency and intensity of activities carried out in a week: high (i.e., approximately one hour of activity per day at an at least moderate intensity activity level), medium (i.e., equivalent to half an hour of at least moderate intensity physical activity on most days), and low (i.e., less than half an hour of moderate physical activity in a week).

The WHO-5 [31,32] scale score ranges from 0 to 25, with 0 representing the worst possible and 25 representing the best possible quality of life. The SF-12v2 mean score is set to 50. 

The GSE-6 [33] measures the level of self-efficacy through six statements on which respondents must indicate the level of agreement and disagreement. The total score ranges between 5 and 25, with a higher score indicating more self-efficacy. 

The total score of the Lubben 6-item Social Network Scale [34,35] is calculated by finding the sum of all items. The score ranges between 0 and 30, with a higher score indicating greater social inclusion. A cut-off of fewer than 12 points of the LSNS-6 is suggested to indicate social isolation, i.e., fewer than two people to perform social integration functions.

The outcome variables answering the second research question are the System Usability Scale (SUS) [37] and the User Experience Questionnaire (UEQ) [37]. The SUS is a Likert scale that includes 10 questions, which study participants ranked from 1 to 5 based on how much they agreed with the statement they were reading. A score of 5 means they agreed completely, and 1 means they strongly disagreed. The average SUS score is 68. Systems recording a score lower than 68 present severe usability problems. The UEQ scale [38] includes eight items: supportiveness, ease, efficiency, clearness, excitement, interest, inventiveness and leading edge. The questionnaire scales cover a comprehensive impression of user experience. Both classical usability aspects (efficiency, perspicuity, dependability) and user experience aspects (originality, stimulation) are measured. The range of the UEQ scales is between −3 (horribly bad) and +3 (extremely good). Values between −0.8 and 0.8 represent a neutral evaluation of the corresponding scale, values > 0.8 represent a positive evaluation, and values < 0.8 represent a negative evaluation.

The third research question was answered through QUAL data collected through the online meetings, during which open-ended questions were asked to stimulate users to think about the weaknesses and strengths of the system and offer suggestions for future digital coaches targeting elders about to retire.

#### 2.2.3. QUANT and QUAL Data Analysis

The QUANT outcome variables were analysed by country (Italy vs. the Netherlands) and by data collection times (T0 vs. T1 and T1 vs. T2) and then compared using the chi-square test for categorical variables and Student’s t-test for continuous variables. A probability value of <0.05 was considered statistically significant. Statistical analysis was performed using SPSS for Win V21.0. For the statistical analysis, only data coming from individuals filling in the questionnaire at the baseline (T0) and at least by the first and/or second follow-up (i.e., T1 and/or T2) and who answered more than ¾ of the questionnaire were considered. Data refer to 62 individuals, 34 from Italy and 28 from the Netherlands. 

QUANT data are reported as mean (±SD) for continuous variables and as absolute frequencies for categorical variables. 

The QUAL data, collected through the online meetings, were audio- and video-recorded after obtaining participants’ signatures on the informed consent form circulated before enrolment in the study and their verbal consent during the online session. Qualitative textual data arising from the transcriptions were analysed using the framework analysis method [39,40,41,42]. The text chunks were associated with codes systematized into a tree chart and combined under main themes. The latter were identified once the consistency within different codes under the same theme had been assessed. Repeated patterns/themes throughout the data set were identified, and a code was associated with every chunk of text. The content areas expressing similar concepts were grouped into mutually exclusive categories associated with codes. Two or more codes were combined, and different codes were sorted into themes and then quotes that were compared on a country basis. For the interpretation, data were rearranged according to the appropriate part of the thematic framework to which it related, and a matrix combining themes and countries was generated [42,43,44,45]. 

Therefore, the analysis started deductively from the aims and objectives of the study embedded in the topic guide but also reflected the respondents’ original observations according to an inductive approach. The parallel and independent analysis by three researchers minimized research bias [46,47,48,49,50]. The QUAL analysis trustworthiness was obtained by scholars’ checks and peer review [51]. 

## 3. Results

### 3.1. Sample Description

The first data collection wave was carried out with 91 people: 53 in Italy (19 older workers and 34 retirees) and 38 in the Netherlands (15 older workers and 23 retirees). Throughout the trial, 13 persons in Italy and 10 in the Netherlands dropped out (see Table 1 below). In Italy and in the Netherlands, the main reasons for dropping out were personal commitments and the lack of interest in the system.

Considering only individuals filling in the questionnaire at the baseline (T0) and at least by the first and/or second follow-up (i.e., T1 and/or T2) and who answered more than ¾ of the questionnaire, the final sample was made of 62 individuals, 34 from Italy and 28 from the Netherlands.

A total of 64.5% of the whole sample consisted of males (see Table 2 below), but with some differences between the national samples. In fact, in Italy, 55.9% of participants were females, while in the Netherlands, they were 10.7%. There was no difference at the country level concerning marital status, i.e., both in Italy and in the Netherlands, married people represented the majority (67.6% and 85.7%, respectively).

The Dutch participants were highly educated; 7 participants had completed between 9 and 13 years of education (25%) and 21 (75%) out of 28 participants had completed more than 13. The Italian participants had a more diverse educational level, with the majority having completed between 9 and 13 years of education (62%), almost a quarter more than 13 years of education (24%), and 15% between 6 and 8 years of education.

In the two countries, retirees represented most of the sample: 66.7% in Italy and 60.7% in the Netherlands. 

### 3.2. The Impact of the AgeWell DC in Improving Healthy Ageing Dimensions

The percentage of participants perceiving their physical condition (SF-12-PCS) as having worsened, i.e., PCS ≤ 50, increased over the experimentation from 19.6% at T0 to 30.6% at T2, while their perception of their mental well-being (SF-12-MCS) remained stable, i.e., the percentage of respondents rating MCS ≤ 42 was 14.3% at T0 and at T2 (see Table 3 below). The pre-post intervention difference is statistically significant for both physical and mental well-being (*p* = 0.002 and <0.001, respectively).

The level of physical activity of participants increased significantly from T0 to T1 and then remained stable up to the end of the trial (*p* = 0.012). It is worth noting that the percentage of people practising a low level of physical activity decreased from 8.2% to 6% after the five trial weeks (the period with the support of the human coach), and it increased again once they started using the system autonomously (from 6% to 8.3%). The difference is statistically significant.

The participants’ mental well-being (WHO-5) and self-efficacy (GSE-6) slightly increased from T0 to T1, but they both decreased from T1 to T2. The level of socialization significantly decreased from T0 to T1 and increased from T1 to T2.

### 3.3. Users’ Experience with the AgeWell Digital Coach

Table 4 shows that out of the six dimensions of the UEQ (attractiveness, perspicuity, efficiency, dependability, stimulation, and novelty), only perspicuity reached a score >0.8, indicating a positive evaluation of the system. All the other dimensions received a bad evaluation by users over the system usage, with small fluctuations from T1 to T2 that only had an increasing trend for “efficiency”, reflecting a slight improvement of the system, while “attractiveness”, “dependability”, and “stimulation” decreased markedly. The system was not rated as “novel” by users from the start of the study onwards.

The usability score of the system (SUS) is 59, that is, 9 points lower than the average score of the scale (68), indicating that the system presents usability problems.

More than half of participants found the DC “moderately/very/extremely useful” after five weeks of usage, but, at the 10th usage week, less than half thought so. Looking at the different system areas of intervention, the percentage of people finding the system useful for “Enriching free time” decreased by 13.1% (from 67.3% to 54.2%), and that of people finding the DC useful for improving mental well-being dropped by 11.4% (from 63.5% to 52.1%). Conversely, participants discovered the usefulness of the AgeWell digital coach for approaching retirement smoothly over time despite initial skepticism; in fact, the percentage of users finding this function useful increased from 50% to 54.2% between T1 and T2.

### 3.4. Strengths and Weaknesses of the System

The QUAL analysis highlighted six main themes: 1. the digital coach’s contents’ strengths; 2. the digital coach’s contents’ weaknesses; 3. the human coach’s role; 4. the digital coach’s technical strengths; 5. the digital coach’s technical weaknesses; 6. users’ suggestions on contents and 7. on technical issues. Themes and sub-themes are briefly shown in Table 5 and analyzed in depth in the text below, supported by quotations extracted from the study participants’ answers, representing examples of opinions common to both Italian and Dutch participants or, conversely, a disagreement and, thus, national specificities. Beyond the information on the respondents’ identification number and country, the quotations from Italian respondents are also followed by information on gender and age (e.g., female, 58), which is not available for the Dutch participants because they did not give their consent to the disclosure of such data.

Concerning the content strengths, trial participants especially recognized the power of the system in five realms:

- Motivating users to increase their physical activity frequency and regularity: “I have had some benefits, for example from walking. Doing it for an hour a day at a steady pace is good for your health and the app makes me fulfill this commitment” (IT1, M, 65, retiree);
- Reminding them to perform activities: “I’m mainly using it as a reminder: it reminds me when I have to do something, some things I honestly do when the app reminds me” (IT3, M, 70, retiree);
- Helping users focus on their own needs (physical, psychological and social): “I have found the app very useful in helping me to focus a little on myself. In fact, I have a tendency to be focused on the needs of others, so someone reminding me “What do you do for yourself?” seems useful” (IT4, F, 65, retiree);
- Stimulating the activities proposed under the realms of mental well-being; some of them thought that several activities were really inspiring, e.g., activities about memories, photographs, yoga and relaxation exercises, or writing down their own qualities: “The app gave me inspiration to call my aunt and ask her to fix and rewrite her recipes. There are so many things that can be taken into account, maybe different from what we do every day” (IT7, M, 69, retiree). This opinion was common, especially among Italian respondents, while Dutch participants mentioned that they liked the proposed activities, but with great variety in the different healthy ageing realms, not only related to emotional well-being but with a wider perspective on the system empowerment capability: “I liked the assignment of being happy with things you have in your home, sending a card to family/friends, the reminder to do some volunteer work, plants” (NL32); “It fits well into my discipline, gives me inspiration and motivation” (NL5);
- Promoting a good retirement process: “It might be helpful for people in danger of falling into a void after retiring” (NL5) and “The digital coach managed to give me the motivation during the day or week to organize myself with new ideas, because a person who suddenly finds himself without work commitments can feel overwhelmed. The app helps you get organized and above all keeps you physically but also psychologically active, because it helps you organize your day, so for me it is useful by readying me for retirement” (IT10, F, 55, older worker).

Concerning the content weaknesses, study participants stressed that:

- Some physical activities proposed by the system were too physically demanding and not fully appropriate for elderly people, and users could not give reasons for not doing the suggested activities, thereby penalizing the overall rate at the end of the week: “On the other hand, the physical activity part that marks the day is not suitable for us. It is not that we do not move around but, for example, on Sunday we usually go for an excursion because we like to walk, but we have done 2 days of heavy gardening, so yesterday we did not feel like walking” (IT43, F, 59, older worker);
- The system was quite rigid, not customizable and not flexible: “What bothers me is the daily activity that is automatically inserted and that I cannot remove” (IT4, F, 65; retiree) and “Planning is tricky, also because I work part-time. (I would prefer not to have a) split between working and not working (retirement). I am not able to fill the day because of work, and my activities like walking dogs and dog training do not fit in (the app)” (NL038);
- The constant pressure the DC put on users by asking them to report the performance was unpleasant: “I would prefer it to be an inspiration tool, not an administration tool” (NL4).

The main technical strength of the system underlined by all participants was the use of videos and video tutorials, as they were considered inspiring and useful for motivating users to put into practice what the videos showed, regardless of the type of activity and the realm it belonged to, e.g., physical activity or psychological well-being, as depicted by the following quotations: “When I was on holiday, I also went to see all the videos that the app offers and they are nice, because they can be stimulating and can make you want to do one thing or the other” (IT12, F, 67, retiree) and “Yoga video clips worked well and fitted [the program of] the gym” (NL09).

Both Italian and Dutch participants stressed the following technical weaknesses:

- Problems in registering/changing the activities they had done. Dutch participants also complained about the long time the platform took to load and the pop-up that mentioned “attempts”;
- Problems with the daily plan, finding it annoying, and preferring a weekly and personalized plan: “I thought it would also be easier and more intuitive to record the activities carried out so that the activity icons in the various sections would be colored. You have to go and click on “I have done it”, but there was one time when it did not record it and I had to go back to it” (IT03, M, 70, retiree);
- The lack of intuitiveness and flexibility: “The logic of the app is not good. It is unpleasant to work with, not user friendly. (This is an) important downside” (NL027);
- The unclearness of graphs reporting daily activity rates: “Overview of notifications is missing in the app. It is tricky that you cannot fill in anything for the day before. Planning something extra on the day itself is not possible” (NL035);
- The avatar was annoying and boring (especially for Italian users), and it had no added value, especially for Dutch users. The latter found the way it looked and sounded not fun and very negative because the avatar repeated messages many times, and it was not interactive. The voice of the avatar was deemed unpleasant and sometimes boring: “Remarks like ‘doing the dishes and cleaning up works well for me’, reminders and specifically derogatory comments like ‘keep it up’ were fake” (NL014).

Suggestions coming from the experimentation mainly concern system contents and technical aspects. Regarding the contents, the users suggested to:

- Personalize activities as much as possible, including by adding other activities such as reading, volunteering and nutrition: “The reading activity does not seem so much specified by the app, maybe less in summer, but in winter it would be better to add it and maybe the app should be diversified on a seasonal basis, in my opinion” (IT9, M, 61, retiree), and “To add other activities”, e.g., volunteering (NL10), nutrition (NL04), gym (NL036);
- Plan periodical meetings with the human coach: “I suggest that every now and then there is a confrontation with the coach, that is, a contact or a video call or a physical meeting (…) I believe that a tool of this kind can be useful, but it must be interspersed with personal relationships, not even with videoconferencing, rather private talk, once with a psychologist, once with a trainer, another time with an expert of any another thing, because it is the relationship that gives us the opportunity to grow, to improve, in my opinion, in terms of both physical and psychological well-being” (IT52,F, 69, retiree);
- Explaining what every proposed activity is useful for in order to further motivate the user to do it: “I could not lend myself to really doing those categories or activities, when I do not know what it is useful for. What does it bring me?” (NL11).

Concerning technical issues, users proposed to:

- Make the app more intuitive: “If there was some more automatic and intuitive function, it would be better, because you always have to go there, see if it works, wait for it to turn and load” (IT03, M, 70, retiree);
- Launch activities with movies and videos to motivate people to perform the activities: “It would be a good idea to start with a movie clip (that) begins with explaining why I should do this activity” (NL11);
- Connect the app activity plan to the smartphone agenda: “The app should link to the agenda on your phone” (ID4).

## 4. Discussion

The novelty of the study lies in testing the impact of a digital coach in motivating elders aged 55 and older in transition to retirement, an overlooked group of users, to adopt healthy ageing lifestyles through several activities proposed via four functionalities addressing healthy ageing components, i.e., physical activity, mental well-being, and socialization and retirement.

### 4.1. Towards Tailored and Attractive Digital Coach Systems

QUANT findings showed that the use of the AgeWell digital coach improved participants’ level of physical activity, mental well-being, and self-efficacy during the first 5 weeks of experimentation, when users were supported by the human coach. It is nevertheless worth noting that after the human coach stopped his/her activity, this positive effect continued only for the physical activity, but not for the mental well-being and self-efficacy. On the one hand, these QUANT results suggest that the digital coach systems can stabilize fluctuations in the physical activity performed by older adults before and after retirement [10,11]. On the other hand, the users’ desire to have periodical meetings with the human coach, evidenced by the QUAL results, may suggest that, for this generation of older adults, it is still important to have the mediation of a human coach to also effect their mental well-being, which is exposed to risk during the retirement process [3,13]. This suggests that the current version of digital health coaching systems cannot fully replace human-led interventions and may not be as effective as those led by human coaches [14,15,16,17,18].

Moreover, QUANT findings tell us that the level of socialization among participants worsened as the trial proceeded during both the human and the digital coach-driven phase. A possible explanation of this result may lie in the fact that, although the number of opportunities for social contacts increased thanks to the periodical distance meetings with the human coach and other users, the virtual nature of these contacts was not so gratifying and satisfying as to improve the perception of the quality of participants’ social ties. In fact, the trial took place between May and October 2021, when many social restrictions were still in force due to the COVID-19 pandemic, and elders participating in the experimentation might have suffered from the medium-term effects of two years of physical distancing. As a result, they might need personal contact with peers to experience meaningful relationships, as in the pre-pandemic period. In fact, although the QUAL findings show that several participants, especially in Italy, appreciated the function of the digital coach stimulating them to keep in contact with friends and relatives, this was not sufficient to improve participants’ capability of increasing the number or the quality of social ties. The existing literature agrees on the fact that technology can improve many dimensions of social connection among older adults, e.g., by communicating and sharing not only pictures and media but also emotions and states of mind and by reading news and books [52,53,54]. 

This highlights the need to design digital coaching systems that are capable of doing this and accordingly improve socialization among older adults. 

Furthermore, both QUANT and QUAL results showed that user experience with the system was not positive in either country. In fact, users found the system unintuitive, not user-friendly and not flexible. Dutch users were particularly attentive to the technical aspects, which is probably due to a greater diffusion of digital systems in this country and to greater digital health literacy among its senior citizens compared to their Italian counterparts, which led them to have higher expectations regarding digital coach systems. Moreover, users did not like the appearance and the attitude of the avatar, which was considered rigid and excessively directive, especially by Italian respondents.

In addition to what Kettunen, Kari and Frank [22] suggested, i.e., that digital coaching devices must be tailored and easy to learn to be attractive to older adults, the above indicates that a key point for the digital coach to be accepted by this target population is the capability of being flexible, funny, interesting, and attractive without being too directive. A characteristic common to older adults in retirement, in fact, is the willingness to be masters of their own life and time, which means being in the condition of choosing what to do with full autonomy in real and digital activities alike [55].

Furthermore, the increased number of users who appreciated the function aimed at making the transition to retirement easier over the experimentation time frame suggests the need for an app that can stimulate healthy ageing during this existential change. This also implies the need for further studies targeting this population group and their experience with digital coaches. The latter should be based on customizable avatars, which is one of the most critical issues that emerged in other studies as well [56,57,58].

In light of the above, the improvement in system usability could also enhance the impact on individuals’ health and well-being.

### 4.2. Strengths and Limitations

The strength of the study is the adoption of a mixed-method approach, which allowed the interpretation of apparently contradictory findings highlighted by QUANT and QUAL tools.

Although the QUAL results highlighted the capability of the system to stimulate older adults to behave in a way more oriented to healthy ageing principles, the numerous technical issues might have confounded the users’ perception of the contents and dimmed the full potential of the app in helping elderly people smoothly cross the line of retirement. This represented a significant limitation of the study, together with the low number of participants, whose recruitment was hindered by the COVID-19 pandemic only in Italy and The Netherlands. Moreover, it was hard to engage older workers in the trial because they had more time constraints than retirees, who represented the majority of the sample. In addition, as a limitation of the study design, the lack of a control group, as well as the length of the trial, did not permit us to go in depth into the effectiveness of the system. All these limitations did not allow for a generalization of results.

Therefore, future large sample studies are encouraged to assess the impact and the effectiveness of digital coach systems in promoting healthy ageing within this elderly population group. Finally, the use of a usable and tailored digital coach may have a positive impact not only on older individuals but also at the company level if we consider older adults near retirement as older workers from a work and human capital perspective [59]. In fact, although there is no difference in productivity between younger and older workers, the latter have more absenteeism than the former [60], representing a huge loss in productivity, as hugely emerged during the COVID-19 outbreak [61]. Although the relationship between age and sickness absence among older workers is not well understood [62], there is a certain concern about it, considering the massive amount of the workforce that is ageing [63]. 

## 5. Conclusions

This study showed that a digital coaching program can be a valuable tool to stimulate older adults to adopt healthy lifestyles while they cross the line of retirement, especially by triggering physical activity. On the contrary, mental well-being and socialization remain the most difficult healthy ageing dimensions to be addressed by digital coach technology. Thus, there is still a need for the support of a human coach in attaining positive mid-term outcomes in these realms. Within this study, some empirical evidence about the characteristics that a coaching system must possess have been highlighted: flexibility, to address the needs of this specific target; being interesting and attractive, to stimulate proactivity and healthy ageing habits during this existential change; and offering a polite communication mode exchange, since elders in retirement want to maintain control over their life and time. Indeed, the level of personalization of such coaching systems remains the golden key to aligning the health program to the physical, cognitive and social status of the intended target. In addition, it can significantly impact the quality of the user-system interaction, usability, and acceptability, as well as enhance adherence to the intervention. Even if technology-based interventions such as coaching systems are an emerging field in the healthcare domain, it seems that there is still a lack of evaluations to discover the added value of this innovation. 

## Figures and Tables

**Table 1:** Overview of participants per country.

Country	Recruited Participants	Drop-Out Pre-Trial	Drop-Out during the Trial	Total Drop-Outs	Invalid Questionnaires	Total Participants
Italy	53	7	6	13	6	34
The Netherlands	38	1	9	10	0	28
Total	91	8	15	23	6	62

**Table 2:** Sample description by country.

	All (n = 62)	Italy (n = 34)	The Netherlands (n = 28)	*p*
	N (%)	N (%)	N (%)	
Gender				
Female	22 (35.5)	19 (55.9)	3 (10.7)	<0.001
Male	40 (64.5)	15 (44.1)	25 (89.3)	
Marital status				
Married/in couple	47 (75.8)	23 (67.6)	24 (85.7)	0.342
Single	5 (8.1)	3 (8.8)	2 (7.1)	
Divorced/separated	6 (9.7)	5 (14.7)	1 (3.6)	
Widowed	4 (6.5)	3 (8.8)	1 (3.6)	
Educational level				
Primary school (1–5 years of education)	0 (0.0)	0 (0.0)	0 (0.0)	-
Secondary school (6–8 years of education)	5 (8.1)	5 (14.7)	0 (0.0)	-
High school (9–13 years of education)	28 (45.2)	21 (61.8)	7 (25.0)	<0.001
Bachelor’s degree (more than 13 years of education)	29 (46.8)	8 (23.5)	21 (75.0)	<0.001
Working condition				
Older workers	23 (36.1)	12 (33.3)	11 (39.3)	0.629
Retirees	39 (63.9)	22 (66.7)	17 (60.7)	

**Table 3:** Impact of the AgeWell digital coach on the healthy ageing dimensions over time.

Healthy Ageing Dimensions	T0 (N = 62)	T1 (N = 52)	T2 (N = 49)	*p* (T0 vs. T1)	*p* (T0 vs. T2)
	N (%)	N (%)	N (%)		
SF-12v2					
PCS ≤ 50, n (%)	11 (19.6)	12 (23.1)	15 (30.6)	0.002	0.055
MCS < 42, n (%)	8 (14.3)	6 (11.5)	7 (14.3)	<0.001	0.001
Level of physical activity (IPAQ-SF)				0.012	0.142
Low	5 (8.2)	3 (6.0)	4 (8.3)		
Medium	25 (41.0)	19 (38.0)	17 (35.4)		
High	31 (50.8)	28 (56.0)	27 (56.3)		
Mental well-being (WHO-5)	13.4 ± 4.6	13.5 ± 4.5	12.9 ± 3.9	0.545	0.897
Self-efficacy (GSE-6)	15.0 ± 3.4	15.4 ± 2.8	14.8 ± 3.1	0.561	0.473
Lubben < 12, n (%)	13 (22.0)	13 (25.0)	12 (24.5)	<0.001	0.002

**Table 4:** User experience and system usability.

Outcome Variables	T1 At 5 Weeks of System Usage July 2021 (N = 52)	T2 At 10 Weeks of System Usage September (N = 49)
	(±SD)	(±SD)
User experience (UEQ)		
Attractiveness	0.58 ± 1.17	0.54 ± 1.18
Perspicuity	0.91 ± 1.29	1.22 ± 1.02
Efficiency	0.31 ± 1.25	0.50 ± 1.22
Dependability	0.73 ± 0.82	0.48 ± 0.95
Stimulation	0.40 ± 1.25	0.34 ± 1.22
Novelty	0.43 ± 1.27	0.43 ± 1.29
Usability of the virtual coach (SUS)	59.0 ± 14.5	59.6 ± 14.1
	N(%)	N(%)
Impact of the virtual coach in the following activities “Moderately/very/extremely useful”		
Advice on physical exercise to practice at home	34 (65.4)	26 (54.2)
Motivating to get in nature and practice physical activity	36 (69.2)	28 (58.3)
Suggestions for mental well-being	33 (63.5)	25 (52.1)
Suggestions for leisure	31 (59.6)	24 (50.0)
Triggering curiosity and interests	34 (65.4)	27 (56.3)
Suggestions for keeping in contact with friends and relatives	27 (51.9)	22 (45.8)
Suggestions for approaching retirement smoothly	26 (50.0)	26 (54.2)
Enriching my free time	35 (67.3)	26 (54.2)

**Table 5:** Themes and sub-themes that arose from the focus groups with Italian and Dutch users.

Themes	Sub-Themes
1. Digital coach’s contents’ strengths	(a) Improving physical activity
	(b) Reminding
	(c) Helping self to self-focus
	(d) Improving psychological/social well-being
	(e) Enhancing healthy ageing (especially during the transition to retirement)
2. Digital coach’s contents’ weaknesses	(a) Some physical activities are not appropriate for older people
	(b) No flexible/personalized activities
	(c) Too much pressure from the digital coach
3. Human coach’s role	(a) Useful periodical meetings
4. Digital coach’s technical strengths	(a) Video tutorials
5. Digital coach’s technical weaknesses	(a) Problems in registering/changing new activities done
	(b) Planning timeframe
	(c) Lack of intuitiveness and flexibility
	(d) Unclearness of graphs reporting daily activity rates
	(e) The avatar
6. Suggestions on contents	(a) Function/activity personalization
	(b) Periodical meetings with a human coach
	(c) Explaining the objective of the proposed activities
7. Suggestions on technical aspects	(a) Making more intuitive
	(b) Launching activities with movies
	(c) Connecting app activities agenda to smartphone agenda

## Data Availability

The data presented in this study are available in the article itself.

## Supplementary Materials

The following supporting information can be downloaded at: https://www.mdpi.com/article/10.3390/ijerph20054034/s1, Table S1: Digital coach activities per functioning realm.
