# Supplementary material for: The Impact of Digital Coaching Intervention for Improving Healthy Ageing Dimensions among Older Adults during Their Transition from Work to Retirement
Source: Int J Environ Res Public Health. 2023 Feb 24;20(5):4034. doi: 10.3390/ijerph20054034 (PMC10001821; doi:10.3390/ijerph20054034)
Supplement: Supplementary file 1 [file ijerph-20-04034-s001.zip › ijerph-2183731-supplementary.pdf]

Supplementary Materials

Table S1. Digital coach activities per functioning realm

**Physical Activities**	**Mental well-being**			**Socialization**		**Retirement**		
**No sub-realms**	**Mind and body**	**Thought and action**	**Emotions and memories**	**You and the others**	**You and the world**	**Activities for transition**	**Suggestions**	**Infos for transition**
**Cycling**	Connection to Ikigai	To do list	Album of memories	Kindness carries you on!		Competences transmission	Sources of inspiration	Retirees' rights
**Walking**	Thinking and movement	Say it out loud	Be grateful for three things (Diary of gratitude)	Fill up with memories!	A new language!	Planning a meeting	Good news!	Who can help you in the transition to retirement: trade-unions
**Gardening**	Count! Breathe!	Ask-Do!	Compliment someone!	2 minute of gratitude!	Film-therapy	Pros and cons of the working life	Spend time with loved ones!	App for planning and economic management like the post office app
**Yoga**	Body connections	Take a break from the web!	Think about your success!	How do others see you?	Art feeds!	Plan a party for retirement!	Be strong!	Explore the activities you may do once retired
**Strength training**	Mindfullness	How we make our wishes come true?	Strengthen the relationship with yourself	Make new acquaintances	New flavors	Parting with work		Municipality website: initiatives for retirees

**Dancing**	Eat green!!	Get out of your comfort zone	How do others see you?	Social roles in balance		Imagine and plan your days
**Dish washing**	Free Dance	Stay awake!	The importance of the little things	Follow your models		Decide which social roles to take on
**Ironing**	Living green!	Put yourself first	Joy from the wardrobe	My social network		Make something good for others
**Mowing the lawn**	Positive psychology!	Look round with new eyes!	How many memories in your home	Share one receipt you made helthier		Good intentions
**Golf**	Change way	Same way, different eyes	Self interpretation	What's been going on today?		
**Soft gymnastic**	Take out your voice!	Change your point of view	Be proud of one habit of yours	Increase the quality of your friendship		
**Horse riding**	Be active!	Bite your tongue!				
**Tennis**	Ten minutes of creativity					
**Swimming**						
